# New Possibilities in the Therapeutic Approach to Alzheimer’s Disease

**DOI:** 10.3390/ijms23168902

**Published:** 2022-08-10

**Authors:** Julia Doroszkiewicz, Barbara Mroczko

**Affiliations:** 1 Department of Neurodegeneration Diagnostics, Medical University of Bialystok, 15-269 Bialystok, Poland; 2 Department of Biochemical Diagnostics, Medical University of Białystok, 15-269 Bialystok, Poland

**Keywords:** Alzheimer’s disease, treatment, pharmacological therapy, neuroinflammation

## Abstract

Despite the fact that Alzheimer’s disease (AD) is the most common cause of dementia, after many years of research regarding this disease, there is no casual treatment. Regardless of the serious public health threat it poses, only five medical treatments for Alzheimer’s disease have been authorized, and they only control symptoms rather than changing the course of the disease. Numerous clinical trials of single-agent therapy did not slow the development of disease or improve symptoms when compared to placebo. Evidence indicates that the pathological alterations linked to AD start many years earlier than a manifestation of the disease. In this pre-clinical period before the neurodegenerative process is established, pharmaceutical therapy might prove invaluable. Although recent findings from the testing of drugs such as aducanumab are encouraging, they should nevertheless be interpreted cautiously. Such medications may be able to delay the onset of dementia, significantly lowering the prevalence of the disease, but are still a long way from having a clinically effective disease-modifying therapy.

## 1. Introduction

Alzheimer’s disease (AD) is the most prevalent cause of dementia in the world and a significant burden to the whole healthcare system. It generally develops in patients above the age of 65. Due to an aging society, every year, we observe an increasing number of affected patients. It is predicted that over 50 million people suffer from AD dementia, and this number will triple by 2050. Pathological processes in AD start at least 20 years before the onset of the disease, making it a chronic disorder. The causes of AD are varied and not entirely understood, and they are not properly placed within the aging process. It is believed that both hereditary and environmental factors may contribute to the disease’s etiology. Less than 5% of all cases of AD are genetic, despite the fact that many gene mutations are linked to the condition. While sporadic, late-onset AD (LOAD) is linked to the APOE 4 gene, early-onset AD (EOAD) is caused by mutations in presenilin 1, presenilin 2, and amyloid precursor protein (APP). The slow development of extracellular plaques made of amyloid β and neurofibrillary tangles (NFTs) made of hyperphosphorylated tau are two histological indicators of AD. Therefore, they result in the loss of synapses and neurons.

**Figure 1:**
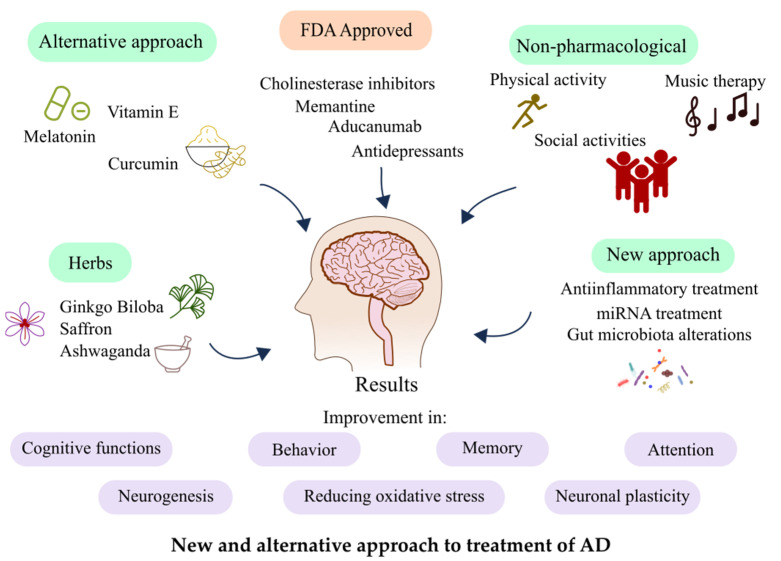
Types of diverse treatment and their results on AD patients.

There is currently no known treatment for AD, and prevention measures are actively being discussed. Presently, the rates for clinical development in AD medication are low and medical research is mostly directed at slowing the progression, not curing the patients. It is related to still not fully known pathophysiology, underlying hallmarks, and heterogeneity of the disease. In this review, we will describe some new and alternative approaches to the treatment of AD, which are demonstrated in Figure 1.

## 2. Pathology of AD

The cardinal pathological hallmarks of the disease cover the accumulation of amyloid β and hyperphosphorylation of tau protein. These pathogenic processes’ aftereffects include neurodegeneration with synaptic and neuronal loss that causes macroscopic atrophy. Mixed pathology—which comprises vascular disease and Lewy bodies—is a common occurrence, especially in elderly people [1]. Multiple cell types express the type 1 transmembrane protein known as the amyloid precursor protein (APP). APP can sequentially cleave through two distinct mechanisms in the central nervous system through gamma and beta-secretases [2]. Aβ40 and Aβ42—two by-products of APP metabolism—are the main components of improperly folded amyloid plaques, which are extracellular accumulations. Due to its increased rate of fibrillization and insolubility, Aβ42 is more common than Aβ40 inside plaques [3]. Aβ may then initiate a chain of events, including neuroinflammation, that cause synapse loss and neuronal death [4].

Tau is a protein that is produced in neurons and which, in healthy cells, has a role in maintaining microtubule stability in the cytoskeleton [5]. It builds up inside nerve cell bodies as these NFTs tangle as a result of hyperphosphorylation. The cellular proteins that are then abnormally interacted with by these tangles are unable to perform their normal activities. The dysfunction of synapses is caused by a decrease in tau binding to microtubules. NFTs are produced in AD patients as a result of increased tau phosphorylation and intracellular tau aggregation caused by an imbalance between tau kinase and phosphatase activity. Finally, the development of NFTs impairs synaptic plasticity [6,7], which damages neuronal cells. Research indicates that Aβ buildup may serve as the catalyst for the downstream process of hyperphosphorylation [8]. There is also proof that toxic tau can increase Aβ production through a feedback loop mechanism [9].

Another recently described hallmark gaining a lot of attention is neuroinflammation. Although the mechanisms promoting AD neuroinflammation have been studied for more than 20 years, they are still not entirely understood. In the inflammatory response in the brain, microglia and astroglia are the crucial members. Microglia can be activated, and act in two types: M1 and M2. M1 phenotype is considered as ‘proinflammatory’ and classical while M2 as ‘anti-inflammatory’ and alternative [10]. Lipopolysaccharide (LPS), IFN, or TNF cause classical activation, which is implicated in pathogen defense mechanisms by the secretion of proinflammatory substances—such as IL-1, TNF, and IL-6—and reactive oxygen species [11]. Conversely, IL-4 and IL-13 cause the M2 phenotype, which releases neuroprotective substances such as TGF, IL-10, and IGF-1 [11]. M2 microglia are able to enhance brain tissue remodeling and repair by regulating inflammation. Interestingly, the M1-to-M2 conversion can happen very quickly [12,13]. Additionally, the proinflammatory milieu created by active microglia around senile plaques encourages the development of the plaques [14,15].

What is more, not only microglia can be activated and influence the course of the disease. Interestingly, activated microglia is capable of inducing A1 astrocytes through secreting Il-1α, TNF, and C1q [16]. According to one hypothesis, astrocytes may gather around plaques and amyloid β, which would encourage their activation. Studies have demonstrated that AD patients’ and animal models’ brains contain active astrocytes [17,18]. Activated astrocytes abandon their neuroprotective functions, simultaneously inducing the death of neurons and oligodendrocytes by releasing proinflammatory cytokines such as TNF-α, IL-6, or IL-12 [16,17,19]. According to Liddelow et al. study, A1 astrocytes secrete a neurotoxin that induces rapid death of neurons and oligodendrocytes [16].

An interesting aspect is connected to iron dyshomeostasis. Many physiological processes in the human body depend on iron, yet as we age, iron is continuously stored in the brain. Early research discovered that Alzheimer’s disease’s cognitive deterioration is directly correlated with iron excess. What is more, both APP and tau protein are connected with iron metabolism [20]. Iron participates in the creation of neurotransmitters, myelination, and antioxidant enzyme activity in the brain [21]. It has been shown that having an excessive amount of iron speeds up the development of neurofibrillary tangles and senile plaques [22]. What is more, a heavy iron diet can cause cognitive deterioration in mice, an increase in aberrant tau phosphorylation in neurons, and inappropriate production of proteins associated with the insulin system. Supplemental insulin can lessen tau phosphorylation brought on by iron [23], proving that iron buildup may interfere with insulin signaling and cause tau hyperphosphorylation.

## 3. Existing Drug Therapies

### 3.1. Cholinesterase Inhibitors

Among established hallmarks of the disease, in the beginning, there were only theories, and the first was the cholinergic one. Acetylcholine (ACh) is a neurotransmitter that is secreted by cholinergic neurons and can be dissected by two cholinesterases (ChEs)—acetylcholinesterase (AChE) and butyrylcholinesterase (BuChE) [24]. The main role of the acetylcholinesterase is in cholinergic neurotransmission by the breakdown of Ach. It is present in both the peripheral and central nervous systems and is associated with learning abilities, cognitive functions, and memory [25]. These actions are conducted by basal forebrain cholinergic neurons (BFCN), which are less present in AD patients because of neuron degeneration [26]. It correlates with the cholinergic hypothesis in which impairment of cognitive functions is obtained through cholinergic neurons degradation and, as a result, disturbances in cholinergic neurotransmission in the disease-affected brain [27]. Brain tissue analysis of AD patients revealed that material obtained from AD patients show neurodegeneration with visible deficits in cholinergic neurons and low levels of acetylcholine. Moreover, the activity of acetylcholine transferase was diminished [28]. It was proven in rodent models that injuring basal forebrain cholinergic neurons results in deficits in maintaining attention [29]. Interestingly, cholinergic synapses were also described as seriously impaired by Aβ molecules [30]. To confirm these revelations, usage of the cholinesterase inhibitors was shown to be capable of improving attention in humans by supporting cholinergic transmission [31]. Moreover, studies show that the cholinergic system is involved in learning processes and memory [32,33,34]. ChEIs work through encouraging the restoration of the cholinergic pathway by inhibiting AChE in the synaptic cleft and, as a result, lowering the hydrolysis of ACh. However, these drugs are mostly used to improve cognitive functions and they do not participate in ‘curing’ the disease.

Traditional ChE inhibitors include donepezil, galantamine, and rivastigmine. Donepezil was the first approved drug in AD treatment and is the first step of the therapeutic approach [35]. However, recent data suggest that prescribing cholinesterase inhibitors to mild cognitive impairment (MCI) patients has no result, or may even have negative consequences on the disease course [36]. Moreover, it has different side effects such as insomnia, gastrointestinal symptoms (nausea, loss of appetite, diarrhea), and those affecting muscles such as cramping and weakness [37,38]. Furthermore, it is estimated that one-third of patients will not show any noticeable results, and the same amount would suffer from side effects, resulting in lowered toleration and disqualifying this drug [4]. Galantamine is the next example of ChEI, which—similarly to donepezil—is prescribed to affect cognitive impairments. It also has comparable side effects, such as gastrointestinal and muscle-related effects [39]. Interestingly, studies showed that continuous rising administration of galantamine might influence the tolerability of the drug [40]. Another substance used for symptomatic treatment of mild to moderate and severe AD is rivastigmine. It is an inhibitor of both AChE and BuChE [41]. It is the only ChEI that can be administered orally and transdermally, with the second method having fewer gastrointestinal side effects [41]. However, the progression of AD cannot be fully stopped or even altered with AChE inhibitors’ help.

### 3.2. Memantine

With the known low tolerability of ChEI, there was a need to find a better substance. Memantine is an agonist to non-competitive N-methyl-D-aspartate (NMDA) receptor that is responsible for transporting neurotransmitter glutamate, which participates in learning and memory [42]. However, excessive amounts of glutamate are considered excitotoxic to neurons; therefore, memantine allows the execution of positive functions of glutamate with a simultaneous decrease in negative ones. Unfortunately, there are some side effects, but they are not severe, such as confusion and anxiety [43].

Meta-analyses of clinical trials showed that using memantine in monotherapy significantly improved cognitive function scores, behavior, and global function in comparison to placebo in patients with AD regardless of the severity and lowering behavioral disturbances in moderate-severe AD [44,45]. Additionally, combination therapies with ChEI altered behavioral disturbances. However, only distributing memantine with donepezil showed better results in combination therapy in comparison to ChEI’s monotherapy [44,46].

### 3.3. Aducanumab

Over the past few decades, many treatments targeting the pathology of AD were unsuccessful due to the complexity of the disease. However, in June 2021, the US Food and Drug Administration (FDA) approved a new AD drug: aducanumab. It is a new-generation monoclonal antibody that is selective to Aβ aggregates, which makes it the first drug rooted in the pathophysiology of AD [47]. Moreover, it is only the fifth drug recommended for AD patients [48]. Firstly, animal model studies revealed that this antibody can significantly improve cognition and lower brain pathology by removing Aβ from the brain [49]. Research shows that aducanumab activates microglial phagocytosis, which stimulates the removal of Aβ plaques [49]. Furthermore, it is able to reduce the formation of the oligomers and, as a result, disturbs the aggregation processes, which makes it the most promising treatment for the disease [47,50].

Though aducanumab was approved by the FDA, it was granted with an accelerated approval pathway, which raised many controversies. One such controversy was the inconsistent results of Phase 3 trials [51,52]. Furthermore, the reduction in Aβ aggregates does not correlate with better neuropsychological function [53]. In addition, new research revealed that tau accumulation, rather than Aβ, is a stronger predictor of cognitive decline [54,55]. Another controversy is that, in the beginning, aducanumab was prescribed to all AD patients, despite the fact that the trials only included MCI and mild AD patients [47,56]. Moreover, side effects of using the drug cover amyloid-related imaging abnormalities (ARIA) which manifests as with micro-bleeds or swelling in the brain, but also dizziness, headaches, and nausea [57].

In Europe, European Medicines Agency had advised against granting marketing authorization for the drug in December 2021. The corporation that proposed aducanumab had asked for the agency’s re-examination, but it withdrew the application before this re-examination was complete [58]. Despite the debate, it is undeniable that aducanumab greatly lowers brain levels of Aβ, a symptom of AD. This observation may mark a turning point in the treatment of AD; however, more research and clinical trials are needed.

### 3.4. Antidepressants

Core characteristics of dementia include not only cognitive impairment but also behavioral and psychological symptoms of dementia (BPSD), commonly referred to as neuropsychiatric symptoms of dementia. Up to 90% of individuals who are given a dementia diagnosis are also diagnosed with BPSD over the course of their illness [59]. The main characteristics of BPSD are apathy, depression, anxiety, sleep disturbances, and even psychosis (such as delusions and hallucinations) [60]. These listed symptoms are a real burden not only to the patients but also to their caregivers. While patients report feeling depressed and having a decreased quality of life, caregivers are more likely to suffer from stress, sadness, and possible financial repercussions—such as loss of employment income [59,61].

When speaking of management, there is no one treatment strategy, due to the complexity and variety of causes of BPSD. However, the first step should always be non-pharmacological interventions both for patients and caregivers. They include psychoeducational interventions, offering dementia patients a variety of meaningful activities, frequently depending on patient preferences and functional capacities such as exercises and psychotherapy [59]. Pharmacological intervention may be introduced if non-pharmacological approaches have failed, a patient’s behavior might be a threat to themselves or others, or if the patient is in substantial distress. Firstly, due to its low side effect burden in comparison to other pharmacological therapies, antidepressants are frequently utilized in the treatment of BPSD. Citalopram and escitalopram are the most common, with the second one having fewer side effects such as the risk of QT prolongation [62,63]. Additionally, sertraline might be considered due to it not resulting in cardiac problems [62]. However, the use of typical antipsychotics and benzodiazepines is controversial and not recommended [59,64,65].

## 4. Alternative Approach

### 4.1. Vitamin E

Vitamin E is a part of the group of fat-soluble vitamins with tocopherols and tocotrienols which have antioxidant abilities, and this vitamin has documented anti-inflammatory abilities [66,67]. Therefore, many years ago, vitamin E started to be recommended as an Alzheimer’s disease treatment. Because of its properties, vitamin E is crucial for maintaining brain health. Meta-analyses of vitamin E concentrations in plasma, serum, and CSF show lowered levels of this vitamin in AD patients [68,69]. However, a more recent case-control study depicted that there is no correlation between AD severity and plasma vitamin E levels, which might indicate that decreased vitamin E consumption may not be the cause of decreased plasma antioxidant status; it may be caused by early disease pathology instead [70]. There are studies confirming that consuming food with vitamin E might reduce the risk of developing AD, but this is not the case in APOE ɛ4 allele carriers [71]. Furthermore, another study showed the connection between supplementing vitamin E and reducing the risk of cognitive decline [72]. Although existing studies show beneficial results of vitamin E, there are some depicting no correlation in AD patients [73,74]. Nonetheless, clinical trials were conducted with inconsistent results [75,76]. What is important to remember is that the trials were conducted on low doses of vitamin E because high ones are considered toxic and have been described to have side effects such as fatigue, gastrointestinal cramps, and diarrhea [77]. So far, clinical investigations have obtained unreliable results regarding the impact of vitamin E on the likelihood of developing AD, and more research should be conducted.

### 4.2. Melatonin

The pineal gland is the primary source of the hormone melatonin. It is a multifunctional, circadian-rhythm-regulated substance that has a neuroprotective role in the development of AD because of its anti-inflammatory and antioxidative effects [78,79]. Levels of melatonin decrease with age, after a constant rise from birth until puberty [79,80]. This is considered an important factor in AD development because of the oxidative damage caused in AD brains due to its decrease in cerebrospinal fluid [81]. Sleep disturbances are closely associated with AD progression. It was revealed that healthy subjects have higher levels of melatonin compared to AD patients [79,82]. Moreover, research shows that melatonin significantly reduced the proinflammatory response caused by Aβ plaques [81]. Interestingly, it was proven that the hormone can prevent Aβ accumulation by directly interacting with Aβ [83]. Additionally, melatonin is able to inhibit NF-κB DNA binding activity [84]. Therefore, melatonin’s anti-amyloidogenic and antioxidant characteristics make this molecule a possible AD therapeutic candidate [84]. Some clinical trials were successful considering the prognosis of developing AD. Lower levels of melatonin correlated with the disease [85]. Furthermore, the administration of melatonin to MCI patients was beneficial and significantly slowed the progression to fully visible AD [85,86]. However, these revelations are not yet sufficient to consider melatonin as a drug for the disease.

### 4.3. Curcumin

Curcumin is an herb worth mentioning regarding AD treatment. Turmeric, a spice with a distinctive yellow color that is frequently used in cooking, contains this polyphenol [87]. The anti-inflammatory, anti-tumor, and antioxidant effects of curcumin are well established [88]. The capacity of curcumin to prevent Aβ and tau aggregation in vivo has been shown by studies on the drug in AD [89]. According to a study by Khanna et al. curcumin may also have anti-inflammatory and neuroprotective benefits [90]. The primary mechanism by which curcumin exerts its effects is thought to be through NFkB inhibition, which is achieved by blocking IkB phosphorylation and subsequent NFkB activation [91]. Moreover, curcumin has the ability to block APP-cleaving enzymes such as β-secretase (BACE-1) [92,93].

Overall, studies on animals have shown highly encouraging findings on the physiological and behavioral potentiation of cognition [94,95]. Human research is more scarce, and the results are less reliable, which makes them harder to understand [96,97]. These discrepancies could be caused by variations in the methodology and the population that was studied. The knowledge of curcumin’s promising effects on cognition might be improved by taking into account assessments of significant inflammatory and antioxidant biomarkers, optimal curcumin doses, dietary interactions, and treatment duration. Future research may also benefit from improving curcumin’s bioavailability due to its poor solubility in water [98].

### 4.4. Other Herbs

#### 4.4.1. Ginkgo Biloba

*Ginkgo biloba* (Gb) extract is a natural medicine, obtained from the oldest living tree species, which is known as helpful in cognition management. Gb is well known for centuries in natural treatment, especially in traditional Chinese medicine [99,100]. It has established properties such as amelioration of blood perfusion [101]. In AD, it is described as having neuroprotective abilities covering antioxidative, antiapoptotic, and anti-inflammatory properties, influencing amyloidogenesis and amyloid β aggregation but also modulating phosphorylation of tau protein [102]. Despite the fact that the extracts are frequently advertised as cognitive enhancers, there is no proof that *Ginkgo biloba* leaf extracts improve memory or attention in healthy individuals [103]. Numerous controlled clinical studies have examined the effects of *Ginkgo biloba* extract on dementia. Meta-analysis shows that there are known valuable effects of GB on people with dementia; however, the results are slightly contradictory due to different dosages, trial durations, and patient characteristics among the trials and should be treated with caution [104].

#### 4.4.2. Saffron

It has been discovered that the spice saffron (*Crocus sativus*) may also have many medicinal properties, also improving memory [105]. According to clinical investigations, saffron has potent antioxidant, anti-inflammatory, and anti-amyloidogenic properties [106,107]. A clinical trial carried out on mild-to-moderate AD patients, comparing saffron extract with donepezil, showed comparable results in improving the cognitive functions [105]. Interestingly, saffron showed fewer side effects from the digestive system than cholinesterase inhibitor. Saffron is also said to be helpful in reducing acetylcholinesterase and acting as a protective agent against toxins [108,109]. Another study found that rats given saffron extract for 21 days had considerably increased levels of antioxidant enzymes and lipid peroxidation products and lower plasma levels of corticosterone. According to the study’s findings, saffron may be helpful in reversing oxidative stress damage to the hippocampus brought on by long-term stress and improving learning and memory deficits [109,110]. A different animal model research covered learning, memory loss, and the induction of oxidative stress, and researchers proved that saffron extract can reduce oxidative stress and the deterioration of learning and memory [110]. Furthermore, clinical trials depicted that saffron might have a positive impact on cognitive function and also obtained similar results to donepezil and memantine [111,112,113].

#### 4.4.3. Ashwagandha and Flavonoids

One of the most popular herbs recommended as a brain rejuvenator for AD is ashwagandha, often known as Indian ginseng or winter cherry, highly valued in Ayurvedic medicine. It is recommended as a nerve tonic, energy booster, and general health and longevity improvement [114,115]. Moreover, it has been demonstrated that ashwagandha contains antioxidant and free radical scavenging properties as well as the capacity to maintain a robust immune system [116]. An APP/PS1 mouse model of AD was treated orally with a semi-purified ashwagandha extract to correct behavioral impairments and prevent the formation of Aβ peptides. The liver’s low-density lipoprotein receptor-related protein was the mediator of this therapeutic effect of ashwagandha [117]. Researchers found that therapy with ashwagandha reduced the toxicity of Aβ while also promoting longevity in a *Drosophila melanogaster* AD model [118]. Although ashwagandha has a large body of research on its therapeutic benefits, there is little information on how it might be used clinically to treat cognitive impairment [115,119]. Importantly, there were no harmful effects of Ashwagandha that stood out in the literature reviews. Additionally, numerous commercialized products and patents acknowledged Ashwagandha’s therapeutic function in treating a number of brain illnesses, including AD; nevertheless, there is a dearth of information on the herb’s molecular pathway, and clinical trials for treating these disorders are unreliable and unpromising [120]. Flavonoids are phytochemical substances with potential uses in medicinal chemistry that are found in many plants, fruits, vegetables, and leaves. They have many beneficial characteristics such as antioxidant, anti-inflammatory, and even neuroprotective functions [121]. Recently described, a natural antioxidant present in vegetables and fruits called Kaempferol is believed to prevent the activation of complement C3 protein. What is more, it is also described as able of stopping the generation of A1 astrocytes, which as described earlier are neurotoxic. Interestingly, it prevents against this astrocytes and 3-nitropropionic acid (NPA) which are known proinflammatory factors [122].

### 4.5. Non-Pharmacological Treatment

#### 4.5.1. Physical Activities

Exercise has become a viable treatment option since it can be utilized as an adjuvant therapy before new, potent medications are created. Practice guideline update summary on MCI showed that physical training over a six-month period could help MCI patients’ cognitive function [123]. Numerous studies have shown that physical activity can halt the process of cognitive deterioration [124,125]. What is more, studies show that physical activity offers significant benefits such as reducing and delaying the onset of severe neuropsychiatric symptoms such as depression, confusion, and apathy [126]. In animal studies, exercise has been shown to stimulate neurogenesis and neuronal plasticity, and aerobic fitness also increases blood flow, glucose uptake, and oxygen extraction while enhancing both structural and functional brain reserves [127,128,129]. Moreover, it was stated that brain-derived neurotrophic factor (BDNF), which is linked to memory and learning, can be secreted more rapidly after exercise [130]. Furthermore, studies have shown that seniors with a normal cognitive function who exercise had a lower risk of developing dementia [131]. Moreover, the beneficial effects of physical activity as an intervention therapy are also supported by numerous systematic reviews and meta-analyses [132,133]. The majority of studies vary in terms of research kind, nonstandard interventions, and research design, so these results are not always followed. The effects of exercise on cognitive functions in Alzheimer’s patients are variable, despite studies emphasizing the necessity of exercise, while some have found no favorable relationship between physical activity and cognitive function in AD patients [131].

#### 4.5.2. Social Activities

A lack of interpersonal engagement, or social isolation, is thought to be the main cause of mental and psychosocial stress, which raises the risk of neurological illnesses [134]. It also accelerates the onset of numerous cognitive problems and raises the risk of morbidity and mortality [134,135]. The mouse model of AD has repeatedly shown that social isolation intensifies memory loss [136,137]. Possible mechanisms underlying this impairment connected with isolation and cognitive impairment include the production of Aβ-peptide and the phosphorylation of tau protein [138], an increase in oxidative stress and inflammatory reactions [139] accompanied by the inhibition of anti-inflammatory responses [140], changes in synaptic plasticity (including a reduction inBDNF) [141], and myelination [142]. However, these processes are not yet fully understood [143]. The benefits of BDNF on brain mechanisms are numerous. For instance, it improves neurogenesis, synaptic plasticity, and cognitive abilities [144]. On the other hand cognitive decline in aging is tied to lower BDNF and its receptor tropomyosin-related kinase B (TrkB) expression [145,146]. In postmortem AD brain samples and MCI patients, lower levels of BDNF protein and mRNA were discovered in the hippocampus [147]. In rat models, age-related changes in gene expression and age-related cognitive impairment were both improved by BDNF injection [148]. Maintaining strong social relationships can help people delay the beginning of AD and lower their risk of cognitive decline, according to doctors and clinical studies. It is true that there is a link between participating in social activities frequently and having higher cognitive performance [149,150]. However, little is understood about the processes that underlie social and emotional influence on the course of the disease.

#### 4.5.3. Music Therapy

Another intriguing hypothesis looks at music therapy as a possible treatment for AD. A growing body of research suggests that this type of treatment may help dementia patients with their memory [151]. It is believed that music interacts with the parts of the brain involved in emotions and decision-making; however, the precise mechanism is still not entirely understood. This has allowed for the identification of some potential mechanisms underlying this behavior, including sympathetic arousal and dopaminergic circuit activation [151]. The music therapy is performed by professional music therapists or formally certified rehabilitation trainers, along with a caretaker for dementia sufferers. In order to achieve a unique purpose in the relationship of music therapy, a pleasant and friendly setting or atmosphere is established by active music therapy, which involves singing, dancing, or instrument performance, or passive music therapy, which involves listening to live or recorded music [152]. The positive effects of music therapy on cognition, emotion, and behavior in AD patients were confirmed by a systematic review undertaken by Garcia-Casares et al. [153]. Interestingly, after only four sessions, this form of therapy helped AD patients’ despair and anxiety and significantly improved their memory and orientation [154]. However, newer studies do not agree with previous data, and further study is required in this area [152].

## 5. New Approach

### 5.1. Anti-Inflammatory Treatment

Knowing how important neuroinflammation is in AD, new potential markers should be analyzed and discovered for a better understanding of the disease. With better knowledge on pro- and anti-inflammatory cytokines released during the disease, discovering and developing a new drug might be much easier.

Non-steroidal anti-inflammatory drug (NSAID) use has been shown to be protective against AD in epidemiological research carried out in recent years. The usefulness of anti-inflammatory medications in the treatment of AD, however, has generated conflicting and inconsistent results from clinical trials [155,156]. This is probably because the unspecific inhibition of both the proinflammatory and anti-inflammatory phenotypes may not be a successful tactic. An even more focused strategy focuses on proinflammatory cytokines that are released later. According to preclinical studies, an anti-TNF antibody effectively reduced amyloid pathology and tau phosphorylation [157,158]. As shown in clinical pilot research, etanercept—a TNF inhibitor—may enhance language function in a small cohort of AD patients [159]. In mouse models of AD, intraperitoneal treatment of an IL-1 receptor-blocking antibody has also shown potential for improving cognition and reducing tau pathology, but additional clinical trials are required to determine the antibody’s safety and effectiveness in people [160]. 

Microglia are known to sustain neuronal function by removing toxic damage in the very early stages of the AD trajectory. These anti-inflammatory characteristics can be maintained for extended periods of time via interfering with microglial activation. Several teams are working on methods to control microglial activation towards a phagocytic and/or anti-inflammatory phenotype [158]. In preclinical investigations, a number of anti-inflammatory cytokines—such as IL-2, IL-4, and IL-33—have demonstrated the capacity to control microglial activation and reduce AD pathogenesis [161,162,163]. However, the results are not conclusive, these potential therapeutics deserve more investigation, including testing in sizable preclinical investigations. Furthermore, the majority of preclinical research has used viral vectors as a gene expression mechanism, making the direct administration of these cytokines into humans a difficulty. New methods to control these cytokines may be necessary [158,164].

### 5.2. miRNA Treatment

MicroRNAs (miRNAs) are single-stranded, non-coding RNA sequences that typically contain 18 to 22 nucleotides [165]. Translational repression or degradation of target genes is caused by miRNA regulation of target genes by binding to complementary 3′-UTRs of mRNAs. miRNAs are involved in many biological processes, including development, differentiation, proliferation, and apoptosis [166,167]. Moreover, they are crucial for appropriate neuronal development and are involved in neuronal plasticity [168]. Consequently, many human disorders—including AD—are influenced by the dysregulation of miRNAs [169,170]. According to several studies, pathological circumstances alter the miRNA profile [171,172]. The dysregulation of miRNA expression in AD patients is supported by an increasing body of research. Several miRNA targets—including Aβ and tau signaling, inflammation, and apoptosis—have been identified, according to a meta-analysis by Swarbrick et al. However, the majority of miRNA targets are still unknown [173]. Additionally, it was shown that the expression of several miRNAs—including miR-29a and miR-29b-1—which control the production of APP and the beta-site APP-cleaving enzyme 1 (BACE1), was downregulated in AD brains. Furthermore, it has been hypothesized that the loss of these particular miRNAs may help to cause an increase in BACE1 and A levels and the improper development of amyloid plaques in sporadic AD [174,175]. Importantly, altered miRNAs can be seen in patients with MCI diagnoses earlier on as well as in fully developed AD. Knowing that miRNAs can regulate Aβ, tau protein, neuroinflammation, and also synaptic function, researchers started to consider changes in the miRNA profiles as a chance to cure the disease. However, with the present knowledge, miRNA research is rather used to study and confirm the connection and origin of the disease.

### 5.3. Gut-Microbiota Modulation

Investigating the relationship between gut bacteria and the neurological system provides a fresh look at neuroinflammation. Numerous recent studies have investigated the role of the so-called “brain–gut axis” in AD pathology [176]. When compared to healthy controls, research on rodent AD models and AD patients has shown that the gut microbiota of AD patients differs from controls, which is linked to the loss of epithelial barrier integrity and persistent systemic and intestinal inflammation [177,178]. LPS, bacterial amyloid, and other toxins that can change physiological barriers and be linked to systemic inflammation are also produced by the gut microbiota. Despite structural differences between the gut and CNS amyloid, the latter can also trigger increased immune reactions, leading to neuronal amyloid aggregation [176,179,180]. Additionally, LPS release from the gut microbiota in particular can activate microglia, which can disrupt the clearance of amyloid and cause neurotoxicity [176]. According to a study by Kim et al., transferring healthy mouse microbiota into AD models reduced glial activation and the development of Aβ plaques and NFTs [177]. In addition, the prevalence of several Escherichia/Shigella-related proinflammatory taxa was positively connected with elevated blood levels of proinflammatory cytokines in amyloid-positive patients and negatively correlated with *E. rectale*, an anti-inflammatory bacteria [181]. It is interesting to note that probiotic treatment increased levels of the anti-inflammatory cytokines IL-4 and IL-6, while decreasing levels of proinflammatory cytokines IL-1α, IL-1β, IL-2, IL-12, IFN, and TNFα [182]. The summary of the results of therapeutic approaches for AD patients is summarized in Table 1.

## 6. Summary

The treatment of patients is still difficult due to AD’s complexity. Only cholinesterase inhibitors, memantine, or a combination of these drugs are currently approved therapies for AD. The AD therapy options, however, continue to be supportive and symptomatic without affecting the long-term outlook. Memantine and cholinesterase inhibitors are two examples of medications that enhance cognition and alertness, respectively, without affecting the lifespan or general course of AD dementia. In addition, numerous novel medications have failed larger Phase 3 trials because they did not reach efficacy endpoints, despite the early promise of many of them.

Existing approaches are not enough while speaking of the cure to the disease but also as slowing agents. Approved drugs have limitations and side effects while alternative and non-pharmacological treatment are useful in single symptoms management without complex view. Moreover, they sometimes depict contradictory meta-analysis results and they vary depending on the person.

The complex pathologic causes of AD, as well as our incomplete understanding of the connections between the various pathways involved in AD development and subsequent neurodegeneration, and the potential ineffectiveness of currently available agents, are all major contributors to the high failure rate of AD therapies under development. Therefore, better understanding of the disease is crucial to develop the medication. Future treatments may enhance presently available medications and reduce the evolution of AD pathology or even the symptoms of the disease. They may also be able to delay or even stop the development of AD symptoms in people who are at high risk for the condition. It is likely that targeting numerous pathways will be necessary for effective treatment, even though combining cholinesterase inhibitors with memantine has had mixed outcomes in the treatment of AD. As a result, more studies looking at plausible agent combinations should be carried out.

## Figures and Tables

**Table 1:** Table summarizing the influence of different types of therapy on AD patients.

Type of Therapy	Influence										Author
	Cognitive Functions	Attention	Learning	Memory	Aβ Plaques	Agitation	Proinflammatory Response	Progression of AD	Emotions	Side Effects	
Cholinesterase inhibitors	↑	↑	↑	↑						insomnia, gastrointestinal symptoms (nausea, loss of appetite, diarrhea), muscle cramping and weakness	[31,32,33,34]
Memantine	↑		↑	↑						Confusion, aniety	[42,43,44,45]
Aducanumab	↑				Removal, disturbing accumulation					micro-bleeds, swelling in the brain, dizziness, headaches nausea	[49]
Antidepressants						↓			↑	Citalopram: risk of QT prolongation	[62,63]
Vitamin E	↑									Overdosage: fatigue, gastrointestinal cramps, diarrhea	[72]
Melatonin					Prevent accumulation			↓		ND	[83,85,86]
Curcumin	↑				Prevent accumulation					ND	[89,94,95]
*Ginkgo biloba*	↑				↓					ND	[102]
Saffron	↑		↑	↑						ND	[109,110]
Ashwaganda					↓toxicity					ND	[118]
Physical activities	↑									ND	[124,125]
Social activities	↑									ND	[144,148]
Music therapy	↑			↑					↑	ND	[151,153]
Anti-inflammatory treatment	↑				↓pathology					ND	[157,158,160]
Gut microbiota alterations					Stopping development					ND	[177]

ND—Not described.

## Data Availability

Not applicable.
